# Theabrownin Alleviates Colorectal Tumorigenesis in Murine AOM/DSS Model via PI3K/Akt/mTOR Pathway Suppression and Gut Microbiota Modulation

**DOI:** 10.3390/antiox11091716

**Published:** 2022-08-30

**Authors:** Hoi Kit Matthew Leung, Emily Kwun Kwan Lo, Hani El-Nezami

**Affiliations:** 1 School of Biological Sciences, University of Hong Kong, Pokfulam, Hong Kong 999077, China; 2 Institute of Public Health and Clinical Nutrition, School of Medicine, University of Eastern Finland, FI-70211 Kuopio, Finland

**Keywords:** colorectal cancer, AOM/DSS model, theabrownin, gut microbiota

## Abstract

Colorectal cancer (CRC) is one of the most common and fatal cancers worldwide, yet therapeutic options for CRC often exhibit strong side effects which cause patients’ well-being to deteriorate. Theabrownin (TB), an antioxidant from Pu-erh tea, has previously been reported to have antitumor effects on non-small-cell lung cancer, osteosarcoma, hepatocellular carcinoma, gliomas, and melanoma. However, the potential antitumor effect of TB on CRC has not previously been investigated in vivo. The present study therefore aimed to investigate the antitumor effect of TB on CRC and the underlying mechanisms. Azoxymethane (AOM)/dextran sodium sulphate (DSS) was used to establish CRC tumorigenesis in a wild type mice model. TB was found to significantly reduce the total tumor count and improve crypt length and fibrosis of the colon when compared to the AOM/DSS group. Immunohistochemistry staining shows that the expression of the proliferation marker, Ki67 was reduced, while cleaved caspase 3 was increased in the TB group. Furthermore, TB significantly reduced phosphorylation of phosphatidylinositol 3-kinase (PI3K), protein kinase B (Akt), and the downstream mechanistic target of rapamycin (mTOR)and cyclin D1 protein expression, which might contribute to cell proliferation suppression and apoptosis enhancement. The 16s rRNA sequencing revealed that TB significantly modulated the gut microbiota composition in AOM/DSS mice. TB increased the abundance of short chain fatty acid as well as SCFA-producing *Prevotellaceae* and *Alloprevotella*, and it decreased CRC-related *Bacteroidceae* and *Bacteroides*. Taken together, our results suggest that TB could inhibit tumor formation and potentially be a promising candidate for CRC treatment.

## 1. Introduction

Colorectal cancer (CRC) is the third most commonly diagnosed cancer worldwide and has singularly contributed to 10% of global cancer prevalence in 2020 [1,2]. The 5-year survival rate of CRC drops tremendously from over 50% to slightly above 10% with the progression from stage III to stage IV [3,4]. Given the asymptomatic nature of CRC until advanced stages, CRC patients often miss the critical window for treatment, which causes CRC to be the second most fatal cancer globally as well [2]. Current therapeutic options for CRC mainly involve surgery, radiotherapy, immunotherapy, and chemotherapy. Yet, side effects, heavy costs, and long duration of therapy are detrimental to both patients and healthcare providers [1,4]. Therefore, there is an urgent need for research into an affordable and effective therapeutic option. 

Theabrownin (TB) is a polyphenol oxidized polymer which contains aromatic rings and residues of polysaccharides and proteins [5,6]. It contributes to the taste and brownish color of Pu-erh tea, a dark tea [7,8]. Its production mainly comes from the fungal fermentation of tea polyphenols, theaflavin and thearubigin, by *Aspergillus tubingensis*, *Aspergillus marvanovae*, and *Aspergillus fumigatus* [9,10,11]. Different processes of the production of fermented tea leaves and extraction method were found to influence the structural composition and size of theabrownin [12,13,14]. However, regardless of these different processes, consistent tumor suppressive effects of TB were found among different research utilizing TB from different sources. TB was found to have tumor suppressive effects against multiple cancers, including non-small-cell lung cancer, osteosarcoma, hepatocellular carcinoma, gliomas, and melanoma, through modulating oncogenic pathways, PI3K/Akt, p53/JNK, and NF-κB pathway [15,16,17,18,19,20]. In CRC, TB was recently reported to inhibit cell growth and promote apoptosis in human CRC cells, HT29 [15]. However, in vitro study disregards the complex microenvironment in CRC and the contribution of gut microbiota. 

Tea polyphenols, including catechins, gallic acid, theaflavins, and tannins, have previously been found to improve obesity, glucose tolerance, hepatic lipogenesis, intestinal barrier, and colorectal cancer through modulating gut microbiota [21,22,23,24,25]. Gut microbiota plays an important role in the development of CRC. The elevation of pathogenic bacteria abundance in CRC worsens the intestine with a damaged intestinal barrier, promoting inflammation through bacterial toxins and bacteria infiltration. Gut microbiota alteration is therefore emerging as a robust candidate in CRC treatment. TB has been proven to exert modulatory effects on gut microbiota and promote production of short chain fatty acids (SCFAs) in mice fed with high fat diet [26,27]. However, current studies have only focused on its prohibitory effects against hypercholesterolemia, hyperlipidemia, oxidative, gluconeogenesis, and other obesity-related metabolic dysfunctions [26,27,28,29,30,31]. 

Based on TB’s reported antitumor properties and ability to modulate gut microbiota, the objective of this study was to examine the efficacy of TB in alleviating colorectal tumorigenesis, gut dysbiosis, and the molecular mechanism behind. The azoxymethane/dextran sodium sulphate model (AOM/DSS) was employed as it highly resembles human CRC formation [32,33].

## 2. Materials and Methods

### 2.1. Chemicals and Antibodies

Azoxymethane (AOM) was purchased from Sigma-Aldrich (St. Louis, MO, USA). Dextran sodium sulphate (DSS) was bought from TdB Labs (Uppsala, Sweden). Theabrownin (TB) (>99.3% purity) was obtained from PURENZHIZAO (Yunnan, China). All primary antibodies were obtained from Abcam (Cambridge, UK) and secondary antibodies were obtained from Biorad (Hercules, CA, USA).

### 2.2. Animals and Experimental Setup

BALB/c male mice (6 weeks old) were purchased from Centre for Comparative Medicine Research (CCMR, HKU). The animals were acclimatized and kept in a 12 h light/dark cycle with regulated temperature and humidity. A normal chow diet and water was given ad libitum. The animal experiment was approved by Committee on the Use of Live Animals in Teaching and Research (CULATR) of the University of Hong Kong (CULATR number: 5481-20). After 1 week of acclimatization, mice (n = 8/group) were randomly allocated into three experimental groups, namely control, AOM/DSS, and TB treatment groups. The AOM/DSS model was established as described previously [34]. The AOM/DSS and TB groups were intraperitoneally injected with AOM (10 mg/kg bw);the control group was injected with PBS only. At week 2, the AOM/DSS group and the TB group were given DSS (2.5%) in their drinking water for one week, followed by one week of normal drinking water. This cycle was repeated for two more rounds. The control group received normal drinking water throughout the experimental period. After the last round of DSS period, the treatment period of five weeks began. TB (225 mg/kg bw) or PBS was given by oral gavage (200 μL) to mice every alternative day until week 12. The dosage of TB was chosen based on previous studies [29]. After sacrifice, the colon was measured and cut open longitudinally for tumor count. Part of the colon was cut for histopathological analysis and fixed in 10% formalin, while the rest of the sample was stored at −80 °C for further biochemical analysis. The small intestine, liver, spleen, and fecal samples were weighed and stored at −80 °C.

### 2.3. Histopathological Analysis

The colon specimens were embedded in paraffin and sectioned (5 μm thickness). Slides were deparaffinized and stained with a hematoxylin and eosin (H&E) kit according to the manufacturer’s instructions (BASO, Wuhan, China). The adenoma counts of each section were examined as described by Huang et al. [35,36]. Briefly, the count of adenomas was divided into microadenomas, low-grade macroadenomas, and high-grade macroadenomas. Sirius red staining (Abcam, Cambridge, UK) was performed according to the manufacturer’s protocol in order to evaluate the degree of colon collagen deposition and fibrosis. The degree of colon fibrosis was quantified by calculating the percentage of positive red staining area over the total colon area measured. Sirius red staining (Abcam, Cambridge, UK) was performed according to manufacturer’s protocol to evaluate the degree of colon collagen deposition and fibrosis.

### 2.4. RNA Extraction and Quantitative Real-Time Polymerase Chain Reaction (RT-qPCR)

Total RNA was extracted from colon and tumor tissues with RNeasy^®^ Plus Mini Kit (Qiagen, Germany) according to the manufacturers’ instructions. Reverse transcription was performed with HiScipt II Q-RT SuperMix for qPCR (Vazyme, Nanjing, China) and C1000 thermal cycler (Biorad, Hercules, CA, USA). The mRNA expression level of gene was determined with the use of AceQ qPCR SYBR Green Master Mix (Vazyme) and run on the StepOnePlus Real Time PCR system (Applied Biosystems, Foster City, CA, USA). The primer sequence is provided in Table 1. Relative gene expression was normalized to β-actin and calculated by the 2−∆∆T method.

### 2.5. Immunohistochemistry (IHC) Staining Analysis

Sectioned slides were deparaffinized, then proceeded to heat induced antigen retrieval with sodium citrate (pH 6). Endogenous peroxidase activity was then quenched with 3% H_2_O_2_. Sections were further blocked with CAS-block reagent (Invitrogen, Waltham, MA, USA) for an hour to prevent nonspecific binding, and exposed with primary antibody including Ki67 (Abcam, 1:100) and c-casp3 (Abcam, 1:100) at 4 °C overnight, and with the secondary antibody at room temperature for 1 h. Chromogen reaction with DAB (Abcam, Cambridge, UK) was then performed to visualize the presence of HRP/peroxidase, and the slides were counterstained with hematoxylin. Live images were taken under the light microscope, and the presence of Ki67 and c-casp3 within colorectal tumor was quantified with the ImageJ software (NIH, USA). The histoscore system was adopted based on Fedchenko et al. [2]. The histoscore of IHC was given by multiplying the percentage of positively stained area with the intensity graded from 0, non-stained; 1, weakly stained; 2, moderately stained; and 3, strongly stained.

### 2.6. Western Blot Analysis

Protein was extracted from colon and tumor tissues using a RIPA buffer with protease and phosphatase inhibitor (Sigma-Aldrich, St. Louis, MO, USA) and centrifuged at 13,000× *g*, 15 min at 4 °C to extract protein. The total protein content was determined with DC protein assay (Bio-Rad, Hercules, CA, USA). An equal amount of extracted protein was electrophorized in 10% SDS-PAGE and transferred to polyvinylidene fluoride (PVDF) membrane. Five percent non-fat milk or BSA were used to block the membrane. The membrane was then incubated with primary antibodies overnight at 4 °C, including anti-PI3K (1:1000), anti-p-PI3K (1:1000), anti-Akt1/2/3 (1:1000), anti-p-AKT1/2/3 (1:1000), anti-mTOR (1:10,000), and anti-cyclin D1 (1:10,000) (Abcam, Cambridge, UK). Secondary antibodies with goat anti-rabbit IgG (H+L) HRP conjugate or goat anti-mouse IgG (H+L) HRP conjugate (Biorad, CA, USA) were used to incubate the membrane afterwards. Imaging of protein bands was visualized with enhanced chemiluminescence reagents (Biorad, CA, USA) under the ChemiDox XRS+ imaging system (Biorad, CA, USA). The intensity levels of the bands are measured with ImageJ software (NIH, Bethesda, MD, USA) and normalization was conducted with reference to the intensity of β-actin.

### 2.7. 16s rRNA Sequencing of Fecal Microbiome

Fecal samples obtained on sacrifice day were used for studying the fecal microbiome. Microbial DNA was extracted using QIAamp^®^ PowerFecal^®^ Pro DNA kit (Qiagen, Hilden, Germany) according to manufacturer instructions. The establishment of sequencing library and Illumina MiSeq sequencing was performed by Novogene Co. Ltd. (Beijing, China). The V3-V4 region of bacterial 16s rRNA gene was amplified with primers, 341F (CCTAYGGGRBGCASCAG) and 806R (GGACTACNNGGGTATCTAAT). In brief, the sequencing libraries were constructed with NEBNext^®^ DNA Library Prep Kit. The constructed library was then sequenced with Illumina platforms. The obtained raw data was analyzed with FLASH (V1.2.7) and filtered by QIIME (V1.7.0) for high quality clean tags. Filtered tags were compared with the reference database, using the UCHIME algorithm to remove chimeras in order to obtain effective tags. Sequences were obtained from Uparse software (V7.0.1090) analysis of these effective tags. Sequences with similarity higher than 97% were clustered into the same operational taxonomic units (OTUs). Representative sequences of OTUs were identified from the 16s SSUrRNA database of SILVA Database to compute the taxonomical annotation. Alpha diversity indices, Chao1, Shannon, Simpson index were calculated using the QIIME software (V1.7.0) and R software (V2.15.3) (Core Team, Vienna, Austria). Beta diversity analysis including Principal Coordinate Analysis (PCoA), Analysis of Similarity (Anosim), and linear discriminant analysis (LDA) of Effect Size (LEfSe) were analyzed by the QIIME software (V1.7.0) (Boulder, CO, USA) and R software (V2.15.3). 

### 2.8. Statistical Analysis

Statistical analysis was performed with GraphPad Prism 8.0 (GraphPad software, San Diego, CA, USA). All data are presented as mean ± standard error of mean (SEM). Comparisons of differences between two groups were analyzed with Student’s *t*-tests or Mann–Whitney U tests. Comparisons of differences in more than two groups were analyzed with one way analysis of variance (ANOVA) followed by Tukey’s multiple comparisons test or the Kruskal–Wallis test followed by Dunn’s multiple comparisons test. The Pearson correlation coefficient was employed to compute the correlation between two variables. A *p*-value < 0.05 was recognized as statistical significance.

## 3. Results

### 3.1. TB Suppressed Tumorigenesis in AOM/DSS 

**Figure 1:**
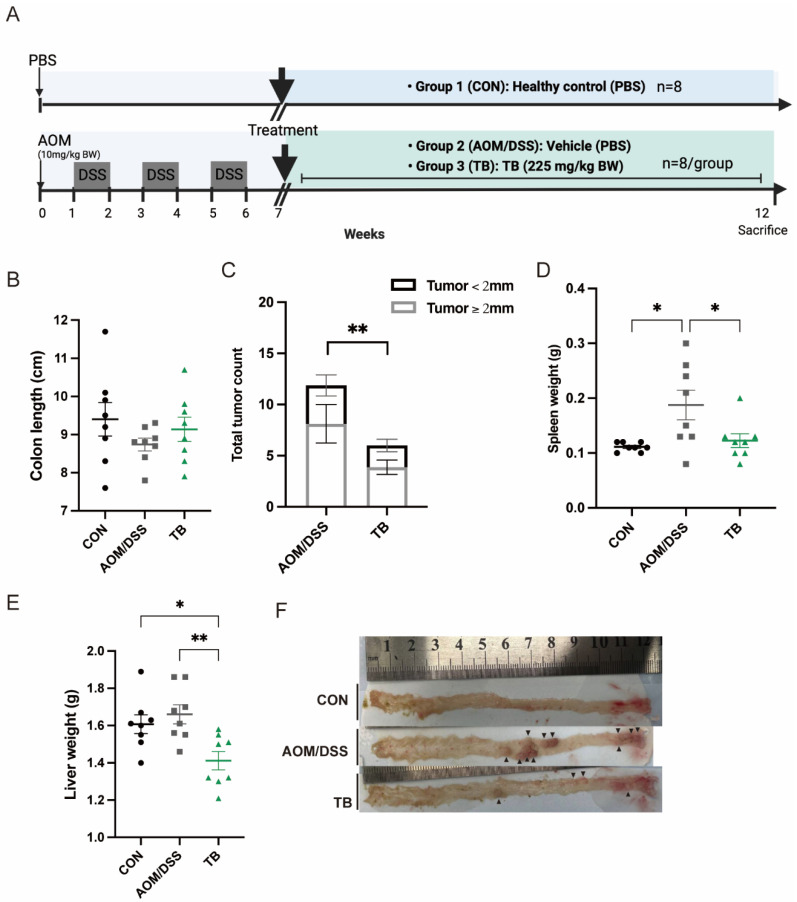
TB suppresses the development of colon tumors in AOM/DSS model. (**A**) Diagram illustrating the AOM/DSS model employed in this study. (**B**). Length of colon in cm. (**C**) Tumor count from colon and its size distribution. (**D**). Spleen weight. (**E**). Liver weight. (**F**). Representative pictures of colon illustrating the presence and location of colorectal tumors, black arrows indicate polyps. n = 7–8 per group. * *p* < 0.05, ** *p* < 0.01.

**Figure 2:**
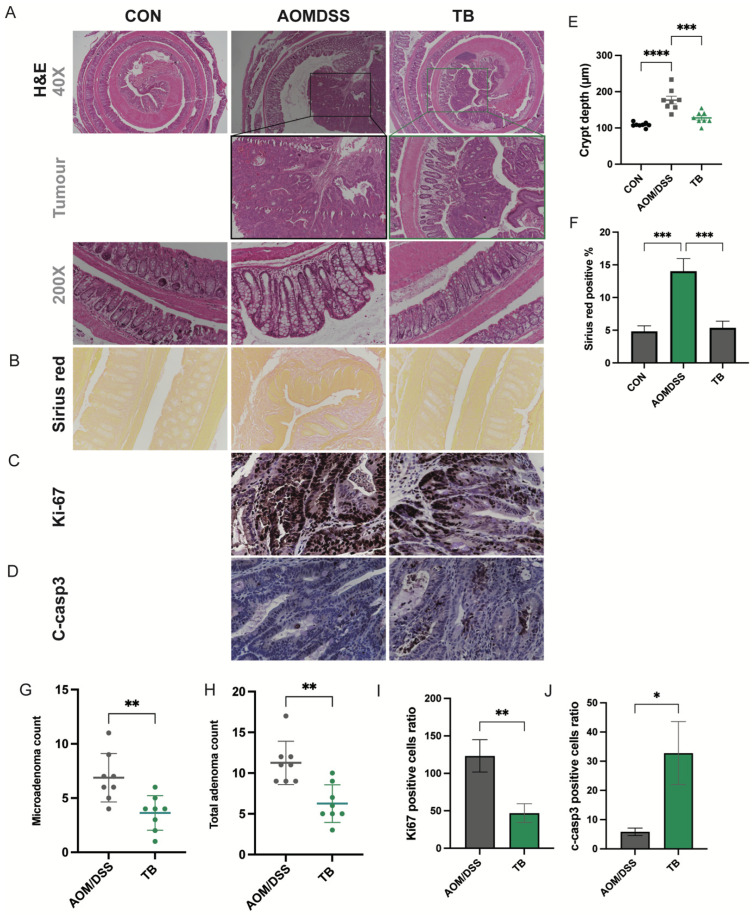
TB suppresses colon cancer progression. Representative photos of H&E staining of (**A**) colon tissue, 40×, tumor areas, 100× and Colon crypt, 200×. (**B**) Representative photos of Sirius red staining of colon tissues. IHC detection of (**C**) Ki67 and (**D**) C-casp3 in tumor areas. (**E**) Colon crypt depth (µm). (**F**) Quantification of Sirius red-positive areas. (**G**) Total adenoma count. (**H**) Total adenoma count per mouse. Positive cell ratio of (**I**) Ki67 and (**J**) c-casp3. n = 7–8 per group * *p* < 0.05, ** *p* < 0.01; *** *p* < 0.001, **** *p* < 0.0001.

In this study, AOM/DSS model were established to explore the effect of TB on colon cancer (Figure 1A). AOM/DSS group has a lower bodyweight gain and higher DAI score compared to the healthy group in week12 (*p* < 0.05) (Figure S1A,B). Although no significant changes were found in colon length (Figure 1B), TB significantly recovered the elevated colonic crypt depth in AOM/DSS mice (*p* < 0.05) (Figure 2A,E). The spleen weight of the TB group was significantly lower than AOM/DSS group (*p* < 0.05) (Figure 1D). Interestingly, the TB group had a lower liver weight when compared to both the healthy and AOM/DSS groups (*p* < 0.05) (Figure 1E). 

The tumor incidence rate induced by AOM/DSS in this study was 100%. The total tumor count was significantly lowered with TB treatment (*p* < 0.05) (Figure 1C,F). TB significantly reduced the total adenoma and microadenoma count in the colon (*p* < 0.05) (Figure 2B,F,G). Both low-grade and high-grade macroadenoma counts were lower in the TB group than in the AOM/DSS group. (Figure S2). Furthermore, Sirius red staining showed a significant increase in percentage of positive area in the AOM/DSS group when compared to control. This indicates the deposition of collagen and the presence of more severe colon fibrosis. TB treatment significantly decreased the Sirius red stained area when compared to AOM/DSS alone (*p* < 0.05), which suggests it has an anti-fibrotic effect. IHC was then performed to understand if TB suppresses tumorigenesis through regulating proliferation and inducing apoptosis. The histoscore of the proliferative marker, Ki67, was higher in the TB group than in the AOM/DSS group (*p* < 0.01) (Figure 2C,I), whereas the histoscore of the apoptotic marker, cleaved-caspase 3 (c-casp3), was significantly improved (*p* < 0.05) (Figure 2D,J), indicating the ability of TB to promote the shift from proliferation to apoptosis in colorectal tumor tissue.

### 3.2. TB Exerts Antitumor Properties via Inhibiting PI3K/Akt Pathway

**Figure 3:**
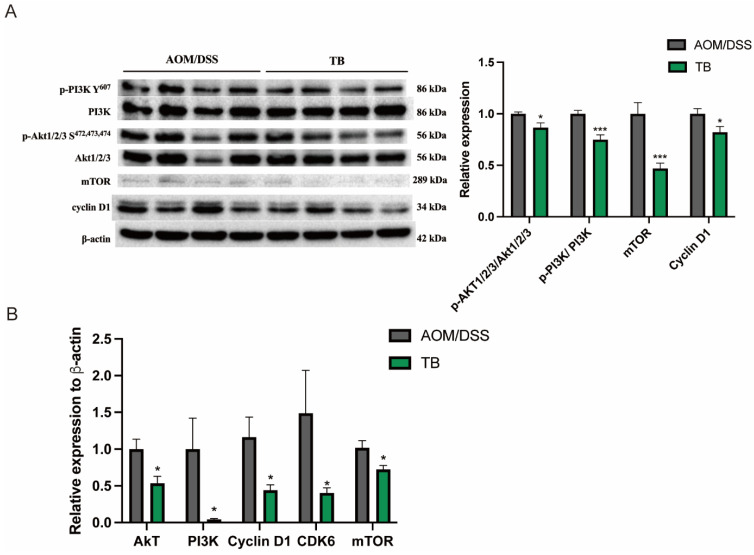
TB inhibits tumor growth through suppressing PI3K/Akt signaling pathway. (**A**) Protein expressions were quantified and normalized against β-actin. (**B**) mRNA expression of Akt, PI3k, Cyclin D1, CDK6, and mTOR was quantified by RT-PCR. n = 7–8 per group * *p* < 0.05, *** *p* < 0.001, compared to AOM/DSS.

To investigate the mechanism by which TB promotes apoptosis, Western blotting was conducted in order to study its impact on the PI3K/Akt pathway, a signaling pathway which regulates apoptosis. Western blot expression showed that TB reduced the phosphorylation of PI3K (*p* < 0.05) and Akt1/2/3 (*p* < 0.05) when compared to AOM/DSS group (Figure 3A). Furthermore, TB significantly suppressed the mRNA levels of Akt, PI3k, Cyclin D1, cyclin dependent kinase (CDK6), mTOR (*p* < 0.05) when compared to AOM/DSS (Figure 3B). The proliferation promoting activator, mTOR and its effector cyclin D1, protein levels were also significantly reduced in the TB group (*p* < 0.05). Taken together, this suggests that the anticancer effects of TB were induced by limiting colorectal tumor proliferation and promoting apoptosis from the suppression of PI3K/Akt pathway.

### 3.3. TB Modified Colon Microbial Composition in AOM/DSS Mice

**Figure 4:**
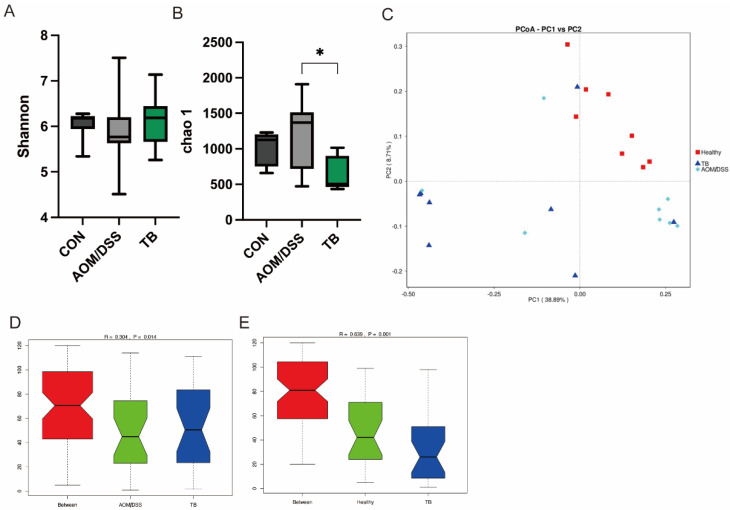
TB regulated gut microbiota diversity. Alpha diversity indices: (**A**) chao1 index, (**B**) Shannon index, (**C**) Weighted Unifac Principal Coordinates Analysis (PcoA). ANOSIM analysis between (**D**) AOM/DSS and TB, (**E**) CON and Tb. n = 7–8 per group. * *p* < 0.05.

The 16s rRNA sequencing was performed to unravel the impact of TB on the microbial composition of AOM/DSS treated mice, with or without TB. Alpha diversity shown by chao1 index was reduced in the TB group (*p* < 0.05) (Figure 4B), while no difference was found in the Shannon index (Figure 4A). In terms of beta diversity, no clear separation was found in the weighted Principal Coordinates Analysis (PCoA) between TB group and AOM/DSS group (Figure 4C). However, the Analysis of Similarity (Anosim) showed that the TB group significantly differed from either the AOM/DSS group or the healthy control group (Figure 4D,E).

**Figure 5:**
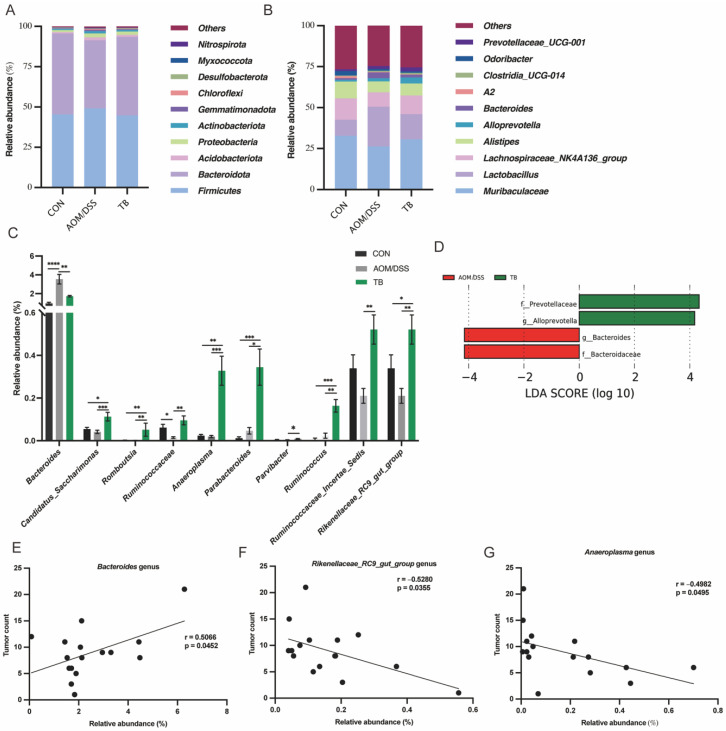
TB altered fecal microbiota abundance. Taxonomical composition at (**A**) phylum and (**B**) genus level. (**C**) Relative abundance in different genus. (**D**) Linear discriminant analysis (LDA) of Effect Size (LEfSe) analysis. Correlation analysis of tumor count and abundance of (**E**) *Bacteroides*, (**F**) *Rikenellaceae_RC9_gut_group*, and (**G**) *Anaeroplasma*. Data are presented as mean ± SEM. * *p* < 0.05, ** *p* < 0.01; *** *p* < 0.001, **** *p* < 0.0001.

At the phylum level, *Firmicutes* and *Bacteroidota* were the predominant phyla in all groups (Figure 5A), accounting for over 90% of the total abundance. However, no difference was found among groups at the phylum level (*p* > 0.05). At the genus level, the fecal microbiota was dominated by *Muribaculaceae*, *Lactobacillus*, *Lachnospiraeceae NK4A136 group* and *Alistipes* (Figure 5B). The abundance of *Candidatus Saccharimonas*, *Romboutsia*, *Anaeroplasma*, *Ruminococcus*, *Ruminoccaceae*, *Rinococcaceae Incertae Sedis*, *Parabacteroides*, *Rikenellaceae RC9 gut group*, and *Parvibacter* genera were significantly elevated by TB compared to the AOM/DSS group (*p* < 0.05) (Figure 5C). Meanwhile, TB significantly reversed the *Bacteroides* abundance increased by AOM/DSS exposure (*p* < 0.05). The linear discriminant analysis (LDA) of Effect Size (LEfSe) analysis was conducted to understand the microbial features and to identify possible biomarkers in the microbial community. TB enriched the SCFA, producing *Prevotellaceae* family and *Alloprevotella* genus (*p* < 0.05), while reducing the CRC-related *Bacteroidceae* family and *Bacteroides* genus (*p* < 0.05) (Figure 5D).

In order to identify the gut microbiota candidate that is associated with TB inhibitory effect on CRC tumorigenesis, the correlation between total tumor count and bacteria abundance was computed among bacteria with significant changes. *Anaeroplasma* genus (r = −0.4982, *p* < 0.05), and *Rikenellaceae_RC9_gut_group* (r = −0.5280, *p* < 0.05) genus were negatively correlated to the total tumor count. On the other hand, the downregulated *Bacteroides* genus (r = 0.5066, *p* < 0.05) was found to be positively correlated to the total tumor count (Figure 5E–G).

## 4. Discussion

In current study, TB has significantly suppressed the colorectal tumorigenesis of AOM/DSS mice by inhibiting proliferation and enhancing apoptosis and gut microbiota modulation. The treatment of TB has significantly reduced the colorectal tumor count, improved microscopic histopathology, and reversed elevated colon crypt depth and colon fibrosis. Our findings are in agreement with Chen et al., which revealed that TB would inhibit the growth of HT-29 human colon cancer cell line [15]. However, because their study focused only on the regulatory effect of TB on the redox system, the effect of TB in regulating colorectal cancer related oncogenic signaling pathways is still unelucidated. In the present study, we found that that the suppression of oncogenic PI3K/Akt/mTOR/cyclin D1 pathway has contributed to the overall anti-CRC effect. Our results assemble with the findings of Wang et al. in which TB alleviates non-small cells lung cancer through the inhibition of the PI3K/Akt pathway and reduction of mTOR and cyclin D1 to achieve apoptosis, autophagy, and cell cycle arrest [17]. The overactivation of the dysregulated PI3K/Akt/mTOR pathway contributes to the progression and proliferation of colorectal cancer cells. The frequency of PI3K/Akt pathway malfunctioning has been observed in over 90% and 80% of human CRC and AOM/DSS model, respectively, indicating it to be an important therapeutic target [37,38]. Mutation-induced overexpression of the PIK3CA and Akt genes in colorectal cancer promotes the activation of PI3K/Akt pathways, and enhances the proliferation and survival of cancer cells while withstanding apoptosis. Several PI3K, Akt, and mTOR inhibitors have entered preclinical and clinical stages in treating colorectal cancer by achieving cell cycle arrest, cell apoptosis and elimination of cell survival signals [39,40,41]. The downstream transcription factor cyclin D1 is positively regulated by Akt and mTOR, in which the degradation of cyclin D1 would accelerate the G1-phase cell cycle arrest in colorectal cancer cells [42,43,44]. In consistency with previous studies, TB was found to be promotive of apoptosis in colorectal cancer with elevated active caspase 3 and reduced proliferative Ki67 quantification in IHC analysis. These data indicate that the suppression of PI3K/Akt/mTOR/cyclin D1 pathway is significant in colorectal tumorigenesis regulation of TB.

In terms of organ weight, TB administration reduced AOM/DSS induced spleen enlargement. Spleen enlargement was commonly observed in AOM/DSS studies as a sign of severity in CRC which was triggered by elevated inflammation and immune functions [45,46]. A similar finding was reported in a study using tea polysaccharides, which reduced AOM/DSS induced spleen enlargement [47]. Intriguingly, the liver weight of the TB group was significantly reduced when compared to both control group and AOM/DSS group. A similar result was observed in the study of Kuang et al. in which TB lowered the liver weight of mice fed with high fat diet suggesting the ameliorative properties of TB in limiting excessive hepatic lipid storage contributed by gut microbiota remodeling [29]. 

Although TB presented strong antitumorigenesis effect in the current study, colitis related symptoms were, unfortunately, not improved. Given that the study design was set as a treatment model, TB was provided only after the establishment of the AOM/DSS model, which might be too late in reversing the shortening in colon length and increase in DAI. The effect of TB with earlier administration on colitis related symptoms is yet to be explored in future studies, given its anti-inflammatory and antioxidative effects observed in previous studies [28,30,48].

Our data have shown that TB differentially impacted the gut microbiota composition. TB reversed the elevated *Bacteroidceae* family and *Bacteroides* genus abundance, which are strongly correlated with the tumor count in the current study. Previous studies reported that *Bacteroidceae* family and *Bacteroides* genus are associated with colorectal cancer and worsened tumor multiplicity and burden in AOM/DSS tumor bearing mice [49,50]. *Bacteroides fragilis* is identified as one of the major pathogens which promotes CRC [51]. In a DSS induced colitis study, elevated abundance of *Bacteroides* by DSS was significantly decreased by Pu-erh tea extracts treatment, and the reversal of gut dysbiosis was achieved [52]. Our finding suggests that TB might be the contributing component in Pu-erh tea which brings the beneficial gut microbiota modulatory effect. From previous studies, *Prevotellaceae* family abundance was reduced upon AOM/DSS treatment [49]. Meanwhile, TB elevated the *Prevotellaceae* family and *Alloprevotella* genus level in rats which consumed a high-fat and high-sugar diet [31,53]. Our data aligns with previous studies that the administration of TB enriched SCFA producing *Prevotellaceae* family and *Alloprevotella* genus in the AOM/DSS model [54]. Short chain fatty acids (SCFA) in the gut, mainly consisting of acetate, propionate, and butyrate, have shown regulatory effects on CRC by alleviating colon inflammation, strengthening the intestinal barrier and regulating the survival of both normal and cancerous colon cells [55,56,57]. Moreover, TB treatment increased the abundance of the beneficial *Romboutsia* genus, which is associated with improved immunity strengthening and anti-inflammatory effects [58,59]. Our study agrees with a previous study that the abundance of *Romboutsia* was significantly increased with ripened Pu-erh tea treatment against DSS induced colitis [40]. Noteworthily, the *Anaeroplasma* genus was strongly correlated to the total tumor count inversely in this study. While the functional effect of the bacteria in CRC is still not well known, the *Anaeroplasma* genus was suggested to be a potential probiotic in treating intestinal inflammation and strengthening mucosal immune response by elevating IgA and TGF-β levels [60,61]. However, to what extent the bacteria contribute to the tumorigenesis regulatory effect remains to be studied further.

## 5. Conclusions

In summary, we found that TB could effectively reduce tumor incidents in AOM/DSS mice. Although TB could not reverse the shortened colon length from AOM/DSS treatment, it reduced the spleen weight, microadenoma and total adenoma count. TB was found to suppress tumor cell proliferation while promoting apoptosis, through its suppression on the PI3K/Akt/mTOR pathway. In addition, TB was found to modify gut microbiota composition. SCFA-producing *Prevotellaceae* family and *Alloprevotella* genus, as well as immunity strengthening and anti-inflammatory *Romboutsia* genus, were significantly upregulated, while pathogenic *Bacteroides* genus were reduced in TB group. Our results confirm the efficacy of TB against CRC development.

## Figures and Tables

**Table 1:** Primer sequences for RT-qPCR.

Genes	NCBI Accession Code	Primer Sequences
Cyclin D1	NM_007631.3	F: TCAAGTGTGACCCGGACTG R: ATGTCCACATCTCGCACGTC
PIK3CA	NM_008839.3	F: CCTCAGCTCTCACCCTCCT R: TTGGTCTCTCTTTCCGCTCAC
AKT1	NM_001165894.1	F: TGAGAAGAAGCTGAGCCCAC R: TAGGAGAACTTGATCAGGCGG
mTOR	NM_020009.2	F: CCGCTACTGTGTCTTGGCAT R: CAGCTCGCGGATCTCAAAGA
Cdk6	NM_009873.3	F: AACCTCTCCTTCGTGAAGACTG R: AGCGATTACATAGTCTGCCCA
β-actin	NM_007393.5	F: AGCCATGTACGTAGCCATCC R: CTCTCAGCTGTGGTGGTGAA

## Data Availability

Data is contained within the article and Supplementary Material.

## Supplementary Materials

The following supporting information can be downloaded at: https://www.mdpi.com/article/10.3390/antiox11091716/s1, Figure S1: (A) Changes of bodyweight and (B) Disease Activity Index (DAI); Figure S2: (A) Macroadenoma count (high grade),(B) Macroadenoma count (low grade).

## References

[1] Xi Y., Xu P. (2021). Global colorectal cancer burden in 2020 and projections to 2040. Transl. Oncol..

[2] Wong M.C., Huang J., Lok V., Wang J., Fung F., Ding H., Zheng Z.-J. (2021). Differences in incidence and mortality trends of colorectal cancer worldwide based on sex, age, and anatomic location. Clin. Gastroenterol. Hepatol..

[3] Rawla P., Sunkara T., Barsouk A. (2019). Epidemiology of colorectal cancer: Incidence, mortality, survival, and risk factors. Gastroenterol. Rev./Prz. Gastroenterol..

[4] Biller L.H., Schrag D. (2021). Diagnosis and treatment of metastatic colorectal cancer: A review. JAMA.

[5] Wu E., Zhang T., Tan C., Peng C., Chisti Y., Wang Q., Gong J. (2020). Theabrownin from Pu-erh tea together with swinging exercise synergistically ameliorates obesity and insulin resistance in rats. Eur. J. Nutr..

[6] Yuan Y., Bai Y., Zhang Y., Wan H., Hu Y., Wu Z., Li X., Song W., Chen X. (2022). Physicochemical and Colon Cancer Cell Inhibitory Properties of Theabrownins Prepared by Weak Alkali Oxidation of Tea Polyphenols. Plant Foods Hum. Nutr..

[7] Wang Q., Peng C., Gong J. (2011). Effects of enzymatic action on the formation of theabrownin during solid state fermentation of Pu-erh tea. J. Sci. Food Agric..

[8] Lin F.-J., Wei X.-L., Liu H.-Y., Li H., Xia Y., Wu D.-T., Zhang P.-Z., Gandhi G.R., Li H.-B., Gan R.-Y. (2021). State-of-the-art review of dark tea: From chemistry to health benefits. Trends Food Sci. Technol..

[9] Wang Q., Gong J., Chisti Y., Sirisansaneeyakul S. (2014). Bioconversion of tea polyphenols to bioactive theabrownins by *Aspergillus fumigatus*. Biotechnol. Lett..

[10] Wang Q., Gong J., Chisti Y., Sirisansaneeyakul S. (2015). Fungal isolates from a pu-erh type tea fermentation and their ability to convert tea polyphenols to theabrownins. J. Food Sci..

[11] Peng C.-X., Liu J., Liu H.-R., Zhou H.-J., Gong J.-S. (2013). Influence of different fermentation raw materials on pyrolyzates of Pu-erh tea theabrownin by Curie-point pyrolysis-gas chromatography–mass spectroscopy. Int. J. Biol. Macromol..

[12] Xu J., Wei Y., Huang Y., Weng X., Wei X. (2022). Current understanding and future perspectives on the extraction, structures, and regulation of muscle function of tea pigments. Crit. Rev. Food Sci. Nutr..

[13] Yamazaki K., Yoshino K., Yagi C., Miyase T., Sano M. (2012). Inhibitory effects of Pu-erh tea leaves on mouse type IV allergy. Food Nutr. Sci..

[14] Xiao Y., Li M., Wu Y., Zhong K., Gao H. (2020). Structural characteristics and hypolipidemic activity of theabrownins from dark tea fermented by single species eurotium cristatum PW-1. Biomolecules.

[15] Chen X., Yuan Y., Wang B., Chen Y., Hu Y., Zhou W., Song W., Wu Z., Li X. (2022). Characterization of theabrownins prepared from tea polyphenols by enzymatic and chemical oxidation and its inhibitory effect on colon cancer cells. Front. Nutr..

[16] Li T., Yan B., Xiao X., Zhou L., Zhang J., Yuan Q., Shan L., Wu H., Efferth T. (2022). Onset of p53/NF-κB signaling crosstalk in human melanoma cells in response to anti-cancer theabrownin. FASEB J..

[17] Wang Y., Yuan Y., Wang C., Wang B., Zou W., Zhang N., Chen X. (2022). Theabrownins Produced via Chemical Oxidation of Tea Polyphenols Inhibit Human Lung Cancer Cells in vivo and in vitro by Suppressing the PI3K/AKT/mTOR Pathway Activation and Promoting Autophagy. Front. Nutr..

[18] Xu J., Xiao X., Yan B., Yuan Q., Dong X., Du Q., Zhang J., Shan L., Ding Z., Zhou L. (2022). Green tea-derived theabrownin induces cellular senescence and apoptosis of hepatocellular carcinoma through p53 signaling activation and bypassed JNK signaling suppression. Cancer Cell Int..

[19] Fu J., Jiang C., Wu M., Mei R., Yang A., Tao H., Chen X., Zhang J., Huang L., Zhao X. (2021). Theabrownin Induces Cell Apoptosis and Cell Cycle Arrest of Oligodendroglioma and Astrocytoma in Different Pathways. Front. Pharmacol..

[20] Jin W., Gu C., Zhou L., Yang X., Gui M., Zhang J., Chen J., Dong X., Yuan Q., Shan L. (2020). Theabrownin inhibits the cytoskeleton-dependent cell cycle, migration and invasion of human osteosarcoma cells through NF-κB pathway-related mechanisms. Oncol. Rep..

[21] Li Y., Gao X., Lou Y. (2019). Interactions of tea polyphenols with intestinal microbiota and their implication for cellular signal conditioning mechanism. J. Food Biochem..

[22] Zhao Y., Zhang X. (2020). Interactions of tea polyphenols with intestinal microbiota and their implication for anti-obesity. J. Sci. Food Agric..

[23] Alves-Santos A.M., Sugizaki C.S.A., Lima G.C., Naves M.M.V. (2020). Prebiotic effect of dietary polyphenols: A systematic review. J. Funct. Foods.

[24] Khairudin M.A.S., Mhd Jalil A.M., Hussin N. (2021). Effects of polyphenols in tea (*Camellia sinensis* sp.) on the modulation of gut microbiota in human trials and animal studies. Gastroenterol. Insights.

[25] Wang S.-T., Cui W.-Q., Pan D., Jiang M., Chang B., Sang L.-X. (2020). Tea polyphenols and their chemopreventive and therapeutic effects on colorectal cancer. World J. Gastroenterol..

[26] Huang F., Zheng X., Ma X., Jiang R., Zhou W., Zhou S., Zhang Y., Lei S., Wang S., Kuang J. (2019). Theabrownin from Pu-erh tea attenuates hypercholesterolemia via modulation of gut microbiota and bile acid metabolism. Nat. Commun..

[27] Wang Y., Zhao A., Du H., Liu Y., Qi B., Yang X. (2021). Theabrownin from Fu brick tea exhibits the thermogenic function of adipocytes in high-fat-diet-induced obesity. J. Agric. Food Chem..

[28] Yue S., Zhao D., Peng C., Tan C., Wang Q., Gong J. (2019). Effects of theabrownin on serum metabolites and gut microbiome in rats with a high-sugar diet. Food Funct..

[29] Kuang J., Zheng X., Huang F., Wang S., Li M., Zhao M., Sang C., Ge K., Li Y., Li J. (2020). Anti-adipogenic effect of theabrownin is mediated by bile acid alternative synthesis via gut microbiota remodeling. Metabolites.

[30] Lei S., Zhang Z., Xie G., Zhao C., Miao Y., Chen D., Zhang G., Liu H., Peng C., Hou Y. (2022). Theabrownin modulates the gut microbiome and serum metabolome in aging mice induced by D-galactose. J. Funct. Foods.

[31] Yue S., Shan B., Peng C., Tan C., Wang Q., Gong J. (2022). Theabrownin-targeted regulation of intestinal microorganisms to improve glucose and lipid metabolism in Goto-Kakizaki rats. Food Funct..

[32] Parang B., Barrett C.W., Williams C.S. (2016). AOM/DSS model of colitis-associated cancer. Gastrointestinal Physiology and Diseases.

[33] De Robertis M., Massi E., Poeta M.L., Carotti S., Morini S., Cecchetelli L., Signori E., Fazio V.M. (2011). The AOM/DSS murine model for the study of colon carcinogenesis: From pathways to diagnosis and therapy studies. J. Carcinog..

[34] Zhao B., Kang Q., Peng Y., Xie Y., Chen C., Li B., Wu Q. (2017). Effect of Angelica sinensis Root Extract on Cancer Prevention in Different Stages of an AOM/DSS Mouse Model. Int. J. Mol. Sci..

[35] Hwang S., Jo M., Hong J.E., Park C.O., Lee C.G., Rhee K.-J. (2020). Protective effects of zerumbone on colonic tumorigenesis in enterotoxigenic Bacteroides fragilis (ETBF)-colonized AOM/DSS BALB/c mice. Int. J. Mol. Sci..

[36] Hamilton S.R., Aaltonen L.A. (2000). Pathology and Genetics of Tumours of the Digestive System.

[37] Pan Q., Lou X., Zhang J., Zhu Y., Li F., Shan Q., Chen X., Xie Y., Su S., Wei H. (2017). Genomic variants in mouse model induced by azoxymethane and dextran sodium sulfate improperly mimic human colorectal cancer. Sci. Rep..

[38] Josse C., Bouznad N., Geurts P., Irrthum A., Huynh-Thu V.A., Servais L., Hego A., Delvenne P., Bours V., Oury C. (2014). Identification of a microRNA landscape targeting the PI3K/Akt signaling pathway in inflammation-induced colorectal carcinogenesis. Am. J. Physiol. Gastrointest. Liver Physiol..

[39] Zhu M., Jin Q., Xin Y. (2021). Recent clinical advances in PI3K inhibitors on colorectal cancer. Die Pharm.-Int. J. Pharm. Sci..

[40] Narayanankutty A. (2019). PI3K/Akt/mTOR pathway as a therapeutic target for colorectal cancer: A review of preclinical and clinical evidence. Curr. Drug Targets.

[41] Bahrami A., Khazaei M., Hasanzadeh M., ShahidSales S., Joudi Mashhad M., Farazestanian M., Sadeghnia H.R., Rezayi M., Maftouh M., Hassanian S.M. (2018). herapeutic potential of targeting PI3K/AKT pathway in treatment of colorectal cancer: Rational and progress. J. Cell. Biochem..

[42] Liu Y., Bi T., Wang Z., Wu G., Qian L., Gao Q., Shen G. (2016). Oxymatrine synergistically enhances antitumor activity of oxaliplatin in colon carcinoma through PI3K/AKT/mTOR pathway. Apoptosis.

[43] Zhang Y., Wang S., Qian W., Ji D., Wang Q., Zhang Z., Wang S., Ji B., Fu Z., Sun Y. (2018). uc. 338 targets p21 and cyclin D1 via PI3K/AKT pathway activation to promote cell proliferation in colorectal cancer. Oncol. Rep..

[44] Zhang X., Min K.-W., Wimalasena J., Baek S.J. (2012). Cyclin D1 degradation and p21 induction contribute to growth inhibition of colorectal cancer cells induced by epigallocatechin-3-gallate. J. Cancer Res. Clin..

[45] Ma F., Sun M., Song Y., Wang A., Jiang S., Qian F., Mu G., Tuo Y. (2022). *Lactiplantibacillus plantarum*-12 Alleviates Inflammation and Colon Cancer Symptoms in AOM/DSS-Treated Mice through Modulating the Intestinal Microbiome and Metabolome. Nutrients.

[46] Jin B.R., Chung K.S., Lee M., An H.J. (2020). High-Fat Diet Propelled AOM/DSS-Induced Colitis-Associated Colon Cancer Alleviated by Administration of *Aster glehni* via STAT3 Signaling Pathway. Biology.

[47] Liu L.Q., Li H.S., Nie S.P., Shen M.Y., Hu J.L., Xie M.Y. (2018). Tea Polysaccharide Prevents Colitis-Associated Carcinogenesis in Mice by Inhibiting the Proliferation and Invasion of Tumor Cells. Int. J. Mol. Sci..

[48] Kim H.-M., Kim E.-M., Lee E.-S., Park N.H., Hong Y.D., Jung J.-Y. (2022). Theabrownin in Black Tea Suppresses UVB-induced Matrix Metalloproteinase-1 Expression in HaCaT Keratinocytes. Biotechnol. Bioprocess Eng..

[49] Zackular J.P., Baxter N.T., Iverson K.D., Sadler W.D., Petrosino J.F., Chen G.Y., Schloss P.D. (2013). The gut microbiome modulates colon tumorigenesis. mBio.

[50] Baxter N.T., Zackular J.P., Chen G.Y., Schloss P.D. (2014). Structure of the gut microbiome following colonization with human feces determines colonic tumor burden. Microbiome.

[51] Cheng W.T., Kantilal H.K., Davamani F. (2020). The mechanism of Bacteroides fragilis toxin contributes to colon cancer formation. Malays. J. Med. Sci. MJMS.

[52] Huang Y., Yang Q., Mi X., Qiu L., Tao X., Zhang Z., Xia J., Wu Q., Wei H. (2021). Ripened Pu-erh Tea Extract Promotes Gut Microbiota Resilience against Dextran Sulfate Sodium Induced Colitis. J. Agric. Food Chem..

[53] Yue S., Peng C., Zhao D., Xia X., Tan C., Wang Q., Gong J. (2022). Theabrownin isolated from Pu-erh tea regulates Bacteroidetes to improve metabolic syndrome of rats induced by high-fat, high-sugar and high-salt diet. J. Sci. Food Agric..

[54] Hu S., Li S., Liu Y., Sun K., Luo L., Zeng L. (2021). Aged ripe Pu-erh tea reduced oxidative stress-mediated inflammation in dextran sulfate sodium-induced colitis mice by regulating intestinal microbes. J. Agric. Food Chem..

[55] Tian Y., Xu Q., Sun L., Ye Y., Ji G. (2018). Short-chain fatty acids administration is protective in colitis-associated colorectal cancer development. J. Nutr. Biochem..

[56] Wang G., Yu Y., Wang Y.Z., Wang J.J., Guan R., Sun Y., Shi F., Gao J., Fu X.L. (2019). Role of SCFAs in gut microbiome and glycolysis for colorectal cancer therapy. J. Cell. Physiol..

[57] Shuwen H., Miao D., Quan Q., Wei W., Zhongshan Z., Chun Z., Xi Y. (2019). Protective effect of the “food-microorganism-SCFAs” axis on colorectal cancer: From basic research to practical application. J. Cancer Res. Clin..

[58] Wang Q., Wang K., Wu W., Lv L., Bian X., Yang L., Wang Q., Li Y., Ye J., Fang D. (2020). Administration of Bifidobacterium bifidum CGMCC 15068 modulates gut microbiota and metabolome in azoxymethane (AOM)/dextran sulphate sodium (DSS)-induced colitis-associated colon cancer (CAC) in mice. Appl. Microbiol. Biotechnol..

[59] de Almeida Brasiel P.G., Luquetti S.C.P.D., Medeiros J.D., do Amaral Corrêa J.O., Machado A.B.F., Moreira A.P.B., Rocha V.N., de Souza C.T., Peluzio M.d.C.G. (2021). Kefir modulates gut microbiota and reduces DMH-associated colorectal cancer via regulation of intestinal inflammation in adulthood offsprings programmed by neonatal overfeeding. Food Res. Int..

[60] Beller A., Kruglov A., Durek P., von Goetze V., Hoffmann U., Maier R., Heiking K., Siegmund B., Heinz G., Mashreghi M.-F. (2019). P104 Anaeroplasma, a Potential Anti-Inflammatory Probiotic for the Treatment of Chronic Intestinal Inflammation. Ann. Rheum. Dis..

[61] Beller A., Kruglov A., Durek P., von Goetze V., Werner K., Heinz G.A., Ninnemann J., Lehmann K., Maier R., Hoffmann U. (2020). Specific microbiota enhances intestinal IgA levels by inducing TGF-β in T follicular helper cells of Peyer’s patches in mice. Eur. J. Immunol..

