# Supplementary material for: Theabrownin Alleviates Colorectal Tumorigenesis in Murine AOM/DSS Model via PI3K/Akt/mTOR Pathway Suppression and Gut Microbiota Modulation
Source: Antioxidants (Basel). 2022 Aug 30;11(9):1716. doi: 10.3390/antiox11091716 (PMC9495753; doi:10.3390/antiox11091716)
Supplement: Supplementary file 1 [file antioxidants-11-01716-s001.zip › antioxidants-1868444-SI.pdf]

Theabrownin alleviates colorectal tumorigenesis in murine AOM/DSS model via PI3K/Akt/mTOR pathway suppression and gut microbiota modulation

Hoi Kit Matthew Leung [1,†], Emily Kwun Kwan Lo [1,†,*] and Hani El-Nezami [1,2,*]

1 *School of Biological Sciences, University of Hong Kong, Pokfulam 999077, Hong Kong, China*
2 Institute of Public Health and Clinical Nutrition, School of Medicine, University of Eastern Finland, FI-70211 Kuopio, Finland
* Correspondence: emilylkk@hku.hk (E.K.K.L.)
†Equally contributed to the manuscript.

Supplementary Figures

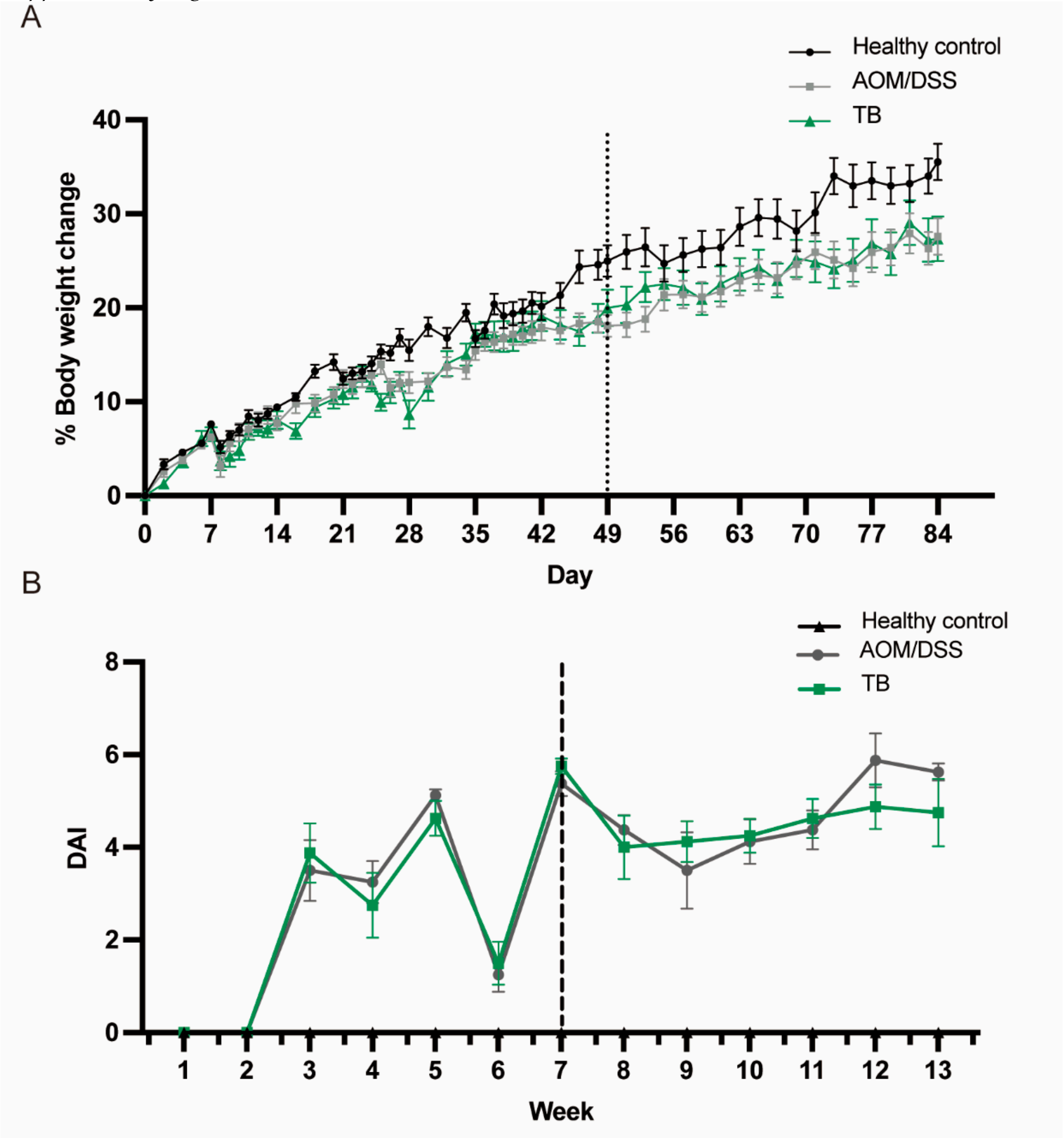

Figure S1. (A) Changes of bodyweight and (B) and Disease Activity Index (DAI)

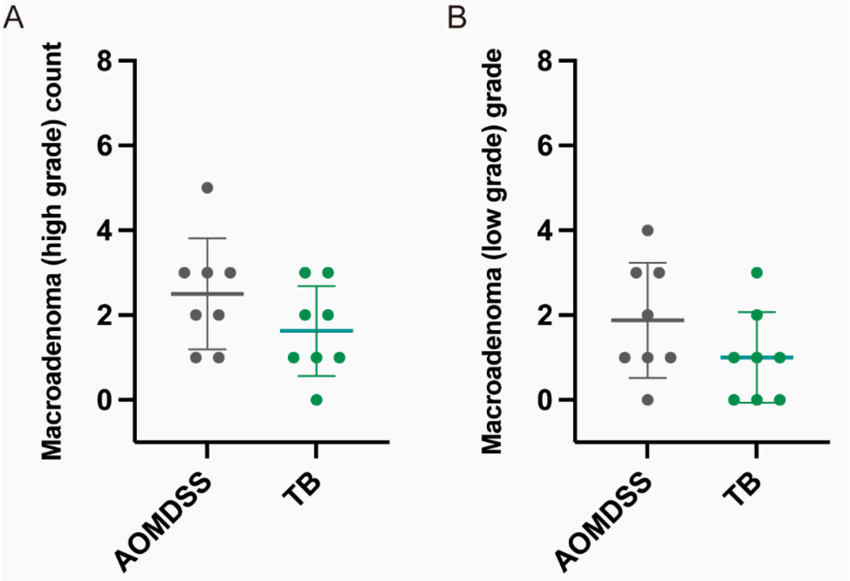

FigureS2. (A) Macroadenoma count (high grade). (B) Macroadenoma count (low grade).

Supplementary Methodology

1. Disease Activity Index (DAI)

The severity of the AOM/DSS induced colitis was monitored with Disease Activity Index (DAI) throughout the experimental period as previously described [1]. In brief, DAI score is a combined score consisted of the measurement of bodyweight loss, stool consistency and stool blood from 0 to 4 (Supp. Table 1). The presence of hemoccult blood was determined with the aid of Hemoccult SENSA testing kit (Hemocue, USA). The scoring of each measurement is as follows:

Supp. Table S1. Disease Activity Index (DAI) scoring.

Score	Weight loss	Stool consistency	Stool blood
0	None	Normal	Negative hemoccult
1	1-5%	Soft but still formed	Negative hemoccult
2	5-10%	Soft	Positive hemoccult
3	10-18%	Very soft; wet	Blood traces in stool visible
4	>18%	Watery diarrhea	Gross rectal bleeding

Reference:

1. Wirtz, S., et al., *Chemically induced mouse models of acute and chronic intestinal inflammation.* Nature protocols, 2017. **12**(7): p. 1295-1309.
